# Supplementary material for: 2019 Survey of Antimicrobial Drug Use and Stewardship Practices in Adult Cows on California Dairies: Post Senate Bill 27
Source: Microorganisms. 2021 Jul 14;9(7):1507. doi: 10.3390/microorganisms9071507 (PMC8304910; doi:10.3390/microorganisms9071507)
Supplement: Supplementary file 1 [file microorganisms-09-01507-s001.zip › Abdelfattah et al. 2021 SM2-Supplementry tables.pdf]

Table S1. Summary of basis and antimicrobial choices for mastitis treatment from responses to questionnaire on antimicrobial drug (AMD) use in adult cows on California dairies during 2019.

Mastitis treatment practices	Missing data	n	Estimate (%)	95% Confidence limits	
				Lower	Upper
**Mastitis: Basis for treatment decision**	**24**	**107**			
Findings of abnormal milk		55	51.4	41.8	60.9
Abnormal milk + Lab testing		33	30.8	22.7	40.4
Abnormal milk + Lab testing + Treat pending test result		19	17.8	11.5	26.3
**Mastitis: Choice of AMD treatment**	**18**	**113**			
Intramammary only		78	69.0	59.8	77.0
Injectable only		6	5.3	2.4	11.4
Intramammary + Oral/Injectables		25	22.1	15.3	30.9
No antimicrobial treatment		4	3.5	1.3	9.2
Mastitis: F**irst choice AMD for intramammary treatment**	**19**	**112**			
Cephalosporins		78	69.6	60.4	77.6
Penicillins		10	8.9	4.8	15.9
Lincosamides		4	3.6	1.3	9.3
Tetracycline		2	1.8	0.4	7.0
None		16	14.2	8.8	22.2
Other (e.g. udder oil)		2	1.7	0.4	7.0
**Mastitis: Second choice AMD for intramammary treatment**	**19**	**112**			
Cephalosporins		20	17.9	11.7	26.2
Lincosamides		16	14.3	8.9	22.2
Penicillins		9	8.04	4.2	14.9
None		65	58.0	48.5	66.9
Other		2	1.7	0.4	7.0
**Mastitis: First choice AMD for oral/injectable treatment**	**20**	**111**			
Cephalosporins		6	5.4	2.4	11.6
Penicillins		4	3.6	1.3	9.3
Sulfonamides		3	2.7	0.9	8.2
Tetracycline		5	4.5	1.9	10.5
None		92	82.8	74.5	88.8
Other		1	0.9	0.1	6.3
**Mastitis:Second choice AMD for oral/injectable treatment**	**19**	**112**			
Penicillins		2	1.8	0.4	7.0
Sulfonamides		1	0.9	0.1	6.2
None		109	97.3	91.8	99.2

Table S2. Summary of basis and antimicrobial drug (AMD) choices for metritis treatment from responses to questionnaire on AMD use in adult cows on California dairies during 2019.

Metritis treatment practice	Missing data	n	Estimate (%)	95% Confidence limits	
				Lower	Upper
**Metritis: Basis for treatment decision**	**30**	**101**			
Clinical presentations (hard calving, retained placenta, vaginal discharge)		42	41.6	32.3	51.6
Physical examination (palpation, rectal temp, treat all fresh cow)		2	2.0	0.5	7.7
Clinical presentaion + examination		57	56.4	46.5	65.9
**Metritis: Choice of AMD treatment**	**25**	**106**			
Bolus/Injectables		61	57.6	47.8	66.7
Intrauterine		22	20.8	14.0	29.7
Intrauterine + Bolus/Injectables		19	17.9	11.7	26.6
None		4	3.7	1.4	9.7
**Metritis: First choice of AMD for Intrauterine treatment**	**27**	**104**			
Cephalosporins		12	11.5	6.6	19.4
Tetracycline		12	11.5	6.6	19.4
Penicillins		3	2.9	0.9	8.7
None		73	70.2	60.5	78.3
Other		4	3.8	1.4	9.9
**Metritis: Second choice of AMD for Intrauterine treatment**	**26**	**105**			
Cephalosporins		4	3.8	1.4	9.9
Tetracycline		2	1.9	0.5	7.5
Penicillins		4	3.8	1.4	9.9
None		95	90.4	83.0	94.0
**Metritis: First choice of AMD for bolus/injectable treatment**	**25**	**106**			
Cephalosporins		46	43.4	34.2	53.1
Penicillins		15	14.1	8.6	22.3
Tetracycline		1	0.9	0.1	6.5
None		36	33.9	25.4	43.6
Others		8	7.5	3.7	14.5
**Metritis: Second choice of AMD for bolus/injectable treatment**	**28**	**103**			
Penicillins		30	29.1	21.1	38.8
Cephalosporins		11	10.7	5.9	18.4
Cephalosporins + Penicillins		2	1.9	0.5	7.6
Tetracycline		1	1.0	0.1	6.8
None		57	55.3	45.5	64.7
Others		2	1.9	0.5	7.6

Table S3. Summary of basis and antimicrobial drug (AMD) choices for lameness treatment from responses to questionnaire on AMD use in adult cows on California dairies.

Lameness treatment practice	Missing data	n	Estimate (%)	95% Confidence limits	
				Lower	Upper
**Lameness: Basis for treatment decision**	**19**	**112**			
Rely on lameness signs		27	24.1	17.0	33.0
Hoof trimmer exam		27	24.1	17.0	33.0
Hoof trimmer exam + Rely on lameness signs		58	51.8	42.4	61.0
**Lameness: Choice of AMD treatment**	**16**	**115**			
Hoof treatment (antibiotic wrap, heel spray, foot bath)		57	49.6	40.4	58.8
Bolus/Injectables		12	10.4	6.0	17.6
Hoof treatment + Bolus/Injectables		44	38.3	29.7	47.6
None		2	1.7	0.4	6.8
**Lameness: First choice of AMD for hoof treatment**	**19**	**112**			
Tetracycline		35	31.3	23.2	40.5
Penicillins		3	2.7	0.8	8.1
Sulfonamides		9	8.04	4.2	14.8
None		52	46.4	37.3	55.8
Others		5	4.4	1.8	10.4
**Lameness: Second choice AMD for hoof treatment**	**20**	**111**			
Tetracycline		7	6.3	3.0	12.8
Penicillins		4	3.6	1.3	9.3
Cephalosporins + Penicillins		1	0.9	0.1	6.3
Sulfonamides		1	0.9	0.1	6.3
None		95	85.6	77.6	91.1
Others		3	2.7	0.8	8.2
**Lameness: First choice AMD for injectable treatment**	**20**	**111**			
Cephalosporins		28	25.2	17.9	34.3
Sulfonamides		11	9.9	5.5	17.1
Penicillins		7	6.3	3.0	12.7
Macrolides		1	0.9	0.1	6.3
Tetracycline		1	0.9	0.1	6.3
None		60	54.1	44.6	63.2
Others		3	2.7	0.8	8.2
**Lameness: Second choice AMD for injectable treatment**	**21**	**110**			
Penicillins		9	8.2	4.3	15.1
Sulfonamides		3	2.7	0.8	8.3
Cephalosporins		3	2.7	0.8	8.3
None		92	83.6	75.3	89.5
Others		3	2.7	0.8	8.2

Table S4. Summary of basis and antimicrobial drug (AMD) choices for pneumonia and postoperative care from responses to questionnaire on AMD use in adult cows on California dairies during 2019.

Pneumonia and post-surgery treatment practice	Missing data	n	Estimate (%)	95% Confidence limits	
				Lower	Upper
**Pneumonia: Basis for treatment decision**	**52**	**79**			
Rely on clinical signs (cough, difficult breathing, nasal disc)		79	100	.	
Not rely on clinical signs		0	0	.	
**Pneumonia: Choice of AMD treatment**	**31**	**100**			
Injectables/bolus: Yes		84	84.0	75.3	90.0
Injectables/bolus: No		16	16.0	10.0	24.7
**Pneumonia: First choice AMD for injectable treatment**	**31**	**100**			
Cephalosporins		32	32.0	23.5	41.9
Penicillins		16	16.0	10.0	24.7
Amphenicols		11	11.0	6.1	19.0
Sulfonamides		4	4.0	1.5	10.3
Macrolides		4	4.0	1.5	10.3
Tetracycline		3	3.0	1.0	9.1
None		28	28.0	19.9	37.7
Others		2	2.0	0.5	7.8
**Pneumonia: Second choice AMD for injectable treatment**	**36**	**95**			
Cephalosporins		8	8.4	4.2	16.2
Penicillins		5	5.3	2.2	12.2
Tetracycline		3	3.2	1.0	9.5
Sulfonamides		1	1.1	0.1	7.3
Amphenicols		1	1.1	0.1	7.3
Fluoroquinolones		1	1.1	0.1	7.3
Macrolides		1	1.1	0.1	7.3
None		71	74.7	64.8	82.5
Others		4	4.2	1.5	10.8
**Post-surgery: Basis for treatment decision**	**76**	**55**			
Routinely after DA[1] or C-Section[2]		10	18.2	9.9	31.1
Rely on veterinarian instruction		28	50.9	37.5	64.2
Routinely after DA + rely on veterinarian		17	30.9	19.9	44.7
**Post-surgery: Choice of AMD treatment**	**37**	**94**			
Injectables/bolus: Yes		46	48.9	38.8	59.1
Injectables/bolus: No		48	51.1	40.9	61.2
**Post-surgery: First choice AMD for injectable treatment**	**39**	**92**			
Penicillins		35	38.0	43.9	64.4
Cephalosporins		6	6.3	2.9	14.0
None		50	54.3	43.9	64.4

Others		1	1.1	0.1	7.5
**Post-surgery: Second choice AMD for injectable treatment**	**39**	**92**			
Penicillins		7	7.6	3.6	15.3

Table S5: Summary of responses for survey about antimicrobial drug use and stewardship practices in adult cows on California dairies during 2019 stratified by herd size, region, and involvement of veterinarian.

Table S6: Description of two clusters identified by using multiple factor analysis and hierarchical clustering of survey responses obtained from 113 conventional dairies in California during 2019 (values with different superscript letters in a row are significantly different (P<0.05).

Components	Characteristics	Cluster 1 (%)	Cluster 2 (%)	P-value
		**N = 104 (92.04)**	**N = 9 (7.96)**	
Herd demography	**Region**	**102**	9	
	NCA[c]	5 (4.90)[b]	2 (22.22)[a]	0.03
	NSJV[d]	48 (47.10)[a]	5 (55.55)[a]	0.62
	GSCA[e]	49 (48.03)[a]	2 (22.22)[a]	0.13
	**Breed**	103	9	
	Holstein	60 (58.25)[a]	3 (33.33)[a]	0.14
	Jersey	2 (1.94)[a]	1 (11.11)[a]	0.102
	Crossbred	5 (4.85)[a]	1 (11.11)[a]	0.424
	Mixed	36 (34.95)[a]	4 (44.44)[a]	0.568
	**Population**			
	Herd size (mean)	1579[a]	685[b]	0.04
	Annual Rolling herd average	11,833[a]	12,250[a]	0.455
	Somatic cell count (median, cells/mL)	150,000[a]	150,000[a]	0.770
Good general practices	Do you harvest colostrum from fresh cows to newborn calves	99	9	
	Yes	95 (95.95)[a]	8 (88.88)[a]	0.333
	No	4 (4.04)[a]	1 (11.11)[a]	0.333
	Do you have a separate pen for recently calved cows	99	9	
	Yes	61 (61.61)[a]	8 (88.88)[a]	0.103
	No	38 (38.38)[a]	1 (11.11)[a]	0.103
**AMD usage information**	**Sources info on AMD used to treat cows**	99	9	
	Include Veterinarian	92 (92.92)[a]	9 (100)[a]	0.410
	Not include Vet, use other sources	7 (7.10)[a]	0 (0)[a]	0.410
	Who decides which oral AMD purchased and stocked	99	9	
	Include Veterinerian	43 (43.43)[a]	3 (33.33)[a]	0.557
	Dairy personnel	56 (56.56)[a]	6 (66.66)[a]	0.557
	Who decides which AMD used to treat sick cows	99	9	
	Include Veterinerian	49 (49.49)[a]	3 (33.33)[a]	0.353
	Dairy personnel	50 (50.50)[a]	6 (66.66)[a]	0.353
Disease management	*Mastitis: Basis for treatment decision*	99	3	

	Abnormal milk + Lab testing + Treat pending test result (1)	19 (19.19)[a]	0[a]	0.400
	Abnormal milk + Lab testing (2)	31 (31.31)[a]	0[a]	0.245
	Finding of abnormal milk (3)	49 (49.49)[a]	3 (100)[a]	0.042
	Mastitis: Treat with AMD	104	5	
	Intramammary and injectable	23 (22.11)[a]	1 (20.00)[a]	0.911
	Intramammary only	74 (71.15)[a]	3 (60.00)[a]	0.592
	Injectable only	5 (4.80)[a]	1 (20.00)[a]	0.145
	No AMD treatment	2 (1.92)[a]	0.00[a]	0.754
	Mastitis: Classes of 1st choice intramammary antibiotic infusion	103	5	
	Cephalosporins	75 (72.81)[a]	3 (60.00)[a]	0.532
	Lincosamides	4 (3.88)[a]	0 (0.00)[a]	0.653
	Penicillins	9 (8.73)[a]	1 (20.00)[a]	0.396
	Tetracyclines	1 (0.97)[a]	0 (0.00)[a]	0.824
	Others	1 (0.97)[a]	0 (0.00)[a]	0.824
	None	13 (12.62)[a]	1 (20.00)[a]	0.631
	Metritis: Basis for treatment decision	96	1	
	Clinical presentations and physical examination	57 (59.37)[a]	0 (0.00)[a]	0.230
	Physical examination only	2 (20.83)[a]	0 (0.00)[a]	0.884
	Clinical presentations only	37 (38.54)[a]	1 (100)[a]	0.210
	Metritis: Choice of AMD treatment	100	1	
	Intrauterine and injectables AMD	19 (19.00)[a]	0 (0.00)[a]	0.628
	Intrauterine AMD only	20 (20.00)[a]	0 (0.00)[a]	0.617
	Injectables AMD only	59 (59.00)[a]	1 (100)[a]	0.410
	No AMD treatment	2 (2.00)[b]	0 (0.00)[a]	0.886
	Pneumonia: Oral bolus/injectable treatment	94	1	
	Yes	79 (84.04)[a]	1 (100)[a]	0.663
	No	15 (15.95)[a]	0 (0.00)[a]	0.663
**Antimicrobial stewardship**	Administration of appropriate AMD, dose, route, and duration	103	4	
	Very important	101 (98.10)[a]	2 (50)[b]	0.001
	Some importance	2 (1.94)[a]	0 (0.00)[a]	0.778
	Not important	0 (0.00)[b]	2 (50.00)[a]	0.001
	Good record keeping on treatments and treatment dates	103	6	
	Very important	100 (97.10)[a]	4 (66.66)[b]	0.005
	Some importance	3 (2.91)[b]	2 (33.33)[a]	0.005
**Producer responses on AMD on dairies**	*Current AMD use practices will make it harder to treat future infections*	*103*	4	
	Strongly agree	15 (14.56)[a]	0 (0.00)[a]	0.410

	Agree	27 (26.21)[b]	3 (75.00)[a]	0.033
	Neutral	33 (32.03)[a]	1 (25.00)[a]	0.766
	Disagree	21 (20.38)[a]	0 (0.00)[a]	0.313
	Strongly disagree	7 (6.79)[a]	0 (0.00)[a]	0.589
	AMD use in livestock does not cause problems in humans	104	4	
	Strongly agree	1 (0.96)[a]	0 (0.00)[a]	0.843
	Agree	7 (6.73)[b]	2 (50)[a]	0.002
	Neutral	28 (26.92)[a]	2 (50)[a]	0.311
	Disagree	40 (38.46)[a]	0 (0.00)[a]	0.118
	Strongly disagree	28 (26.92)[b]	0 (0.00)[a]	0.227
	AMD use in livestock leads to bacterial infections in people	103	4	
	Strongly agree	3 (29.12)[a]	1 (25)[b]	0.022
	Agree	13 (12.62)[a]	1 (25)[a]	0.471
	Neutral	34 (33)[a]	1 (25[a]	0.737
	Disagree	38 (36.89)[a]	1 (25)[a]	0.627
	Strongly disagree	15 (14.56)[a]	0 (0.00)[a]	0.410

Table S7. Responses of California dairy producers (n=75) to survey questions on antimicrobial drug (AMD) use and stewardship practices in adult cows during 2018, and 2019.

Questions	2018 (%)	95% confidence limits		2019 (%)	95% confidence limits		*P*-value
		Lower	Upper		Lower	Upper	
Do you keep a drug inventory log for your dairy? Yes	37.8	26.7	48.8	40.2	28.9	51.6	0.762
Have you submitted any non-routine samples for disease diagnosis? *Yes*	44.4	32.9	55.9	43.8	32.4	55.2	0.941
Do you have a veterinarian-client-patient relationship (VCPR) for this dairy? *Yes*	91.4	84.8	97.9	96.0	91.5	100	0.254
Familiarity with medically important antimicrobial drugs (MIADs)	75.0	64.7	85.3	69.0	58.2	79.7	0.432
Aware that MIADS require a VFD or prescription	97.1	93.2	100	98.6	96.0	100	0.527
Changes that farm made regarding AMD use (*Decreased AMD use*)	39.4	28.1	50.8	36.7	25.3	48.2	0.7458
Have you begun or increased use of alternatives to antibiotics (*Yes*)	30.5	19.9	41.2	27.4	17.2	37.6	0.675
Have you made changes in management to prevent disease spread (*Yes*)	40.5	28.9	52.1	23.94	14.0	33.8	0.035
Farm's antibiotic drug cost as compared to 2018 and earlier							
Decreased	28.2	17.7	38.6	28.2	17.7	38.6	1.00
Increased	23.9	14.0	33.8	15.4	7.0	23.9	0.210
No Change	47.8	36.2	59.5	56.3	44.8	67.8	0.313
Farm's animal health as compared to 2018 and earlier							
Better	33.3	22.2	44.4	44.2	32.6	55.9	0.185
Worse	5.7	0.2	11.3	2.8	1.0	6.7	0.393
No change	60.8	49.3	72.3	52.8	41.1	64.5	0.340
How *very important* the administration of appropriate drug, route, and duration	92.9	87.0	98.9	98.5	95.7	100	0.051
How *very important* good record keeping on AMD treatments	94.3	89.0	99.7	94.3	89.0	99.7	1.00

How *very important* having VCPR	79.7	70.2	89.2	73.2	62.9	83.5	0.367
How *very important* to observe the withdrawal periods and drug residues	97.1	93.2	100	95.7	95.7	90.9	0.648
How *very important* using alternatives to antibiotics	44.2	32.6	55.9	52.8	41.2	64.5	0.310
Producers *strongly agree or agree* that current AMD use will make it harder to treat in future	36.7	25.3	48.2	36.6	25.4	47.8	0.985
Producers *strongly agree* or agree that AMD use in livestock does not cause problems in humans	60.0	48.5	71.4	57.7	46.2	69.2	0.785
Producers *strongly agree or agree* that AMD use in livestock lead to bacterial infections in people that more difficult to treat	5.6	0.2	10.9	15.4	7.0	23.9	0.02
Producers *strongly agree or agree* that any use of AMD may result in infections that are more difficult to treat in future	14.1	5.9	22.1	19.7	10.4	28.9	0.370
Producers *strongly agree or agree* that they are willing to treat animals with alternatives if they are equally effective as AMD	81.6	72.6	90.6	85.7	77.5	93.9	0.517
